# Rehabilitation for Mitochondrial Membrane Protein-Related Neurodegeneration: A Case Study

**DOI:** 10.7759/cureus.50540

**Published:** 2023-12-14

**Authors:** Ömer Faruk Özçelep, Atahan Turhan, Sibel Fi̇dan, Safiye Kandemir

**Affiliations:** 1 School of Physical Therapy and Rehabilitation, Kırşehir Ahi Evran University, Kırşehir, TUR

**Keywords:** rehabilitation, brain iron accumulation, neurodegeneration, functional independence, postural control, balance and coordination, physiotherapy intervention

## Abstract

This case report reports the effects of an 18-week physiotherapy program in children with mitochondrial membrane protein-associated neurodegeneration (MPAN). The study involved two brothers, aged 11 and 12, who had been diagnosed with MPAN. The physiotherapy program was divided into three phases and consisted of 18 weeks of training with a pediatric physiotherapist, including balance, coordination, and strengthening exercises. Muscle strength was assessed using pediatric manual muscle testing, functional balance using the Pediatric Berg Balance Test (PBBT), static balance using the Single-Leg Stance Test, dynamic balance using the Functional Reach Test, postural control using the 5-Time Sit-to-Stand Test, and independence using the Functional Independence Measure for Children (WeeFIM). Positive changes were observed in muscle strength, balance, and independence. After Phase I, PBBT scores (younger sibling +4, 8.1%; older +3, 6.8%) were higher than the minimal clinically important difference (MCID=3.66-5.83). After Phase III, although the PBBT scores improved (younger +2, 4.05%; older +1, 2.3%), the older sibling’s score was not higher than the MCID. Thus, the two children showed visible improvements in both body structure and function, as well as activity and participation levels.

## Introduction

Iron accumulation in the brain, especially in the basal ganglia, frequently leads to neurodegeneration [1]. Mutations in C19orf12 have recently been identified in individuals with a diagnosis of neurodegeneration with brain iron accumulation (NBIA) [2]. The C19orf12 gene, which is localized in mitochondria and the endoplasmic reticulum, encodes a mitochondrial membrane protein. Mutations in this gene have been hypothesized to lead to the disruption of lipid homeostasis within the mitochondria, resulting in the onset of mitochondrial membrane protein-related neurodegeneration (MPAN) [1-3]. Patients diagnosed with MPAN typically experience gait disturbance as a result of lower limb spasticity and dystonia [4]. Other symptoms include parkinsonism, spastic paresis, dysarthria, cerebellar ataxia, behavioral problems, dementia, psychomotor delay, peripheral neuropathy, visual changes such as optic atrophy, and bowel/bladder incontinence [4-6]. MPAN cases exhibit more variability than other NBIA cases, however, in terms of age of onset (between three and 30 years) and rate of progression [7].

There is no rehabilitation protocol in the literature regarding exercise practices for MPAN syndrome. This study aims to reveal the effect of an 18-week therapeutic intervention on balance, coordination, and postural control in MPAN syndrome.

## Case presentation

Two brothers with MPAN, aged 11 and 12, who were born at term with birth weights of 2,000 g (36 weeks) and 3,500 g (40 weeks), respectively, presented to the clinic with a complaint of falling. Muscle weakness, loss of coordination and balance, and gait disturbance were observed during the initial assessment (Table 1).

**Table 1:** Timeline of events related to patient treatment

Events	Date
Recognizing toe walking	2017
Rehabilitation in special clinics	2017–2018
Genetic screening for the family	2020
Rehabilitation in special clinics and hospital	2020–2022
Applying for the hospital	11.20.2022
Phase I	01.06.2023–02.21.2023
Inpatient treatment	07.05.2023–08.20.2023
Phase II home exercises program	09.20.2023–10.20.2023
Phase III	10.23.2023–12.01.2023

The mother experienced her first pregnancy at the age of 25 (150 cm, 55 kg). Jaundice or trauma was not encountered in either child post-delivery. Apart from the premature birth of the younger sibling, no substantial birth difficulties were observed. The younger sibling did not demonstrate any motor developmental delays and acquired head control at three months and sitting at five months. The older sibling began walking at 14 months, whereas the younger sibling started walking at just nine months, without crawling first. At the age of five years, the older brother began experiencing falls, prompting his mother to seek medical attention. The younger brother underwent a check-up due to concerns raised by his teacher regarding vision and reading difficulties during his second year of primary school. When the older brother was seven years old, a physical therapist was consulted due to frequent falls and pes planus. Genetic screening was performed on the entire family, resulting in the detection of related gene damage in both siblings. Vitamin supplements were administered, and rehabilitation was recommended.

Assessments

Body Structures and Functions

Assessments were based on the International Classification of Functioning, Disability and Health Child and Youth Version (ICF-CY). Hip extensor and abdominal muscle strength was evaluated by manual muscle testing (MMT) and scored from 0 to 5. Standard positions and procedures were used [8]. In pediatric research, instrumental maximum voluntary contraction should be preferred over MMT scoring [9]. Quantitative methods, however, have the disadvantage of requiring a specific tool rather than a clinician’s hands [10].

Functional balance was evaluated with the Pediatric Berg Balance Test (PBBT). Reliability testing for PBBT, performed with a sample of 20 children aged five to 15 years with mild to moderate motor impairments, showed good test-retest reliability (intraclass correlation coefficient [ICC] = 0.998) and good interrater reliability (ICC = 0.997) [11]. Validity testing for PBBT, performed with a sample of 30 children aged four to 10 years with spastic cerebral palsy in Gross Motor Function Classification System (GMFCS) Levels I-III, showed a strong correlation between the Pediatric Balance Scale and the self-care (r = 0.73, p < 0.001) and mobility (r = 0.82, p < 0.001) dimensions of the Pediatric Disability Evaluation Inventory [12]. After completing the 12-week program encompassing phases I and II, postural control and static and dynamic balance assessments were included. We assessed static balance using the single-leg stance test and found a standard error of measurement (SEM) of 8.7 s and a minimum detectable change (MDC) of 24.1 s at a 95% confidence level. We also assessed dynamic balance using the Functional Reach Test and postural control using five repetitions of the Sit-to-Stand Test.

Activity and Participation

Participation in activities of daily living (ADL) was evaluated with the Functional Independence Measure for Children (WeeFIM). WeeFIM is easy and efficient to administer and is a valuable predictor of function in children with disabilities. WeeFIM includes 18 items with a 20-minute administration time. As WeeFIM is shorter and quicker to administer, it lends itself to assessment of functional outcome in pediatric rehabilitation. Higher scores represent more independence in participation [13]. Test-retest for the six domains range from r = 0.83 to r = 0.99. Internal consistency (Cronbach’s alpha, or ICC) of the WeeFIM motor and cognitive scales was high (>0.90) and consistent for individual use. Interrater reliability was excellent, with ICC values of 0.98 and 0.93 for the motor and cognitive scales, respectively.

Therapeutic management

The initial phase of the rehabilitation program was structured according to the patients’ main problems. The primary concern was the impairment of balance during regular daily activities, followed by loss of muscle strength in the abdominals and hip extensors. It was identified that implementing exercises aimed at improving balance and coordination while walking was an important step forward [14]. Following these objectives, the Phase I scheme was developed in accordance with Table 2.

**Table 2:** Phase I (0 to six weeks/in clinic) rehabilitation program

Problem identified	Goals	Physiotherapy intervention
Muscle weakness in abdominals and hip extensors	To increase muscle strength and prevent anterior pelvic tilt	Abdominal muscle-strengthening exercises were prescribed to both siblings to perform 10 times per day, 5 days a week. In the supine position, the patients were asked to lie on their knees with the knees flexed. The hip extensors were trained in the prone position on the bed by resisting hip extension.
Loss of balance and coordination	To improve balance and coordination and therefore independence in walking	The following exercises were applied 5 times a week, with 10 repetition once a day: Clapping hands while marching, standing on one leg in front of the mirror, walking in tandem, rhythmic dynamic stabilization, swiss ball exercises (eyes open and closed)

Results of Phase I

Following the initial phase, which involved muscle strengthening and balance training, the children demonstrated improvement in several measures, including the PBBT, WeeFIM scores, and the strength of their abdominals and hip muscles. Notably, the increase in the PBBT score was significantly greater than the MCID reported in the literature for children with cerebral palsy [15]. In the older brother, there was an increase in the ability to stand on one leg (PBBT score from 1 to 2), complete a 360-degree turn (from 3 to 4), and reach forward while standing (from 3 to 4). The younger brother showed an improvement in unsupported standing with one foot forward position (from 0 to 4). The observed 2-point increase in WeeFIM scores for both siblings was attributed to an increase in stair-climbing activity. For detailed findings, please refer to Table 3.

**Table 3:** Results of Phase I rehabilitation program Y: Younger brother, O: Older brother, MCID: Minimal Clinical Important Difference, WeeFIM: Functional Independence Measure for Children

Component	Measurement method	Parameters	Subject	Pre- rehabilitation of Phase I	Post-rehabilitation of Phase I	Change (%)	MCID
Muscle strength	Pediatric manual muscle testing	Abdominals	Y	3	5	+2 (%66)	-
			O	3	4	+1 (%25)	
		Hip extensors	Y	4	5	+1 (%33)	
			O	3	5	+2 (%66)	
Functional balance	Pediatric Berg Balance Test	Total score	Y	49	53	+4 (%8.1)	3.66-5.83
			O	44	47	+3 (%6.8)	
Independence	WeeFIM	Total score	Y	108	110	+2 (%1.6)	-
			O	119	121	+2 (%1.6)	

Phase II (six to 12 weeks/at home) initiative was based on the caregiver's ability and the suitability of home conditions. Exercises were prescribed for home-based practice, as stiffness of the muscles around the feet and ankles in the morning and balance and coordination irregularities were observed. Following clinical conditions and legal regulations, a six-week hiatus was taken, and the objective was to preserve the previous progress through home-based exercises during this period. The home exercise regimen consists of straight leg lifts, side leg lifts, wrist flexor and gastro-soleus muscle stretches, standing up and sitting down repeatedly, and 20 minutes of cycling on an ergometer and light jogging every day.

The younger sibling increased functional independence by 9 points, while the older sibling experienced an increase of 1 point before Phase III. Both siblings experienced a 2-point increase in balance; however, this improvement was below the MCID. As muscle strength was already gained to the desired extent during Phase I and there was no observed loss in relevant muscle strength before Phase III, strengthening exercises were not added to the rehabilitation program. Phase III was designed according to Table 4.

**Table 4:** Phase III (12–18 weeks/in clinic) rehabilitation program

Problem identified	Goals	Physiotherapy intervention
Persistent disturbances in balance and coordination	To increase walking independence by enhancing balance and coordination	The following exercises were performed 5 times a week, once a day, with the specified number of repetitions: Heel to toe raise (20 times), stand up and turn each side (5 times), single-leg stance with chair support initially (10 times), tandem walking (5 m), marching with opposite arm lifts (soldier walking)(5 mm), side lunges (10 times), weight bearing for both sides (10 times), giant step backward (10 times), crossing one leg in front of or behind the other in a continuous manner (5 m)

Results of Phase III

The assessment conducted after Phase III showed that progress had been made in all measures except for the older sibling’s ability to stand on one leg and complete the 5-Time Sit-to-Stand Test. Among these achievements, only the dynamic balance metric for the younger sibling exceeded the minimum clinical significance threshold (Table 5).

**Table 5:** Results of Phase III rehabilitation program Y: Younger brother, O: Older brother, MCID: Minimal Clinical Important Difference, WeeFIM: Functional Independence Measure for Children

Component	Measurement method	Parameters	Subject	Pre- rehabilitation of Phase III	Post-rehabilitation of Phase III	Change	MCID
Static balance	Single-Leg Stance Test	Right	Y	3.9 s	10 s	+6.1	24.1 s
			O	1.5 s	2.45 s	+0.95	
		Left	Y	2.18 s	7.5 s	+5.32	
			O	3.45 s	2.32 s	−1.33	
Independence	WeeFIM	Total score	Y	119	123	+4	-
			O	122	125	+3	
Functional balance	Pediatric Berg Balance Test	Total score	Y	52	54	+2	3.66-5.83
			O	49	50	+1	
Dynamic balance	Functional Reach Test	Total score	Y	18 cm	24 cm	+6	4-11 cm
			O	18 cm	18 cm	-	
Postural control	5 Times Sit-to-Stand Test	Total score	Y	8.01 s	8.91 s	+0.9	2.5 s
			O	10.29 s	9.58 s	−0.71	

## Discussion

This is the first clinical case report to investigate the effects of a physiotherapy and rehabilitation program on body structure, function, and levels of activity and participation in a child with MPAN. The syndrome presents a variety of symptoms, ranging from gait disturbances to vision problems. Implementing a multidisciplinary approach to rehabilitation is therefore crucial in managing the syndrome.

Prevalent physical disorders in cases of MPAN include spasticity, ataxia, muscle weakness, and associated gait issues. In our two cases, there was a crouch gait present due to the shortening of the hamstrings and gastro-soleus [4-6]. Initially, the disease appeared to have a spastic diplegic appearance. It was generally thought by pediatricians at the time that the children may have had cerebral palsy prior to the gene screening. However, based on the electroencephalogram reports and the bilateral involvement of the substantia nigra, red nucleus, and dentate nucleus in both siblings, as well as putamen involvement in the older sibling and globus pallidus in the younger sibling, the presence of brainstem involvement in our patients signifies the differentiation between MPAN disease and classical spastic diplegic cerebral palsy.

One of the important features of the disease is that it can occur in a wide age range (from three to 30 years), as reported in the literature [7]. In our cases, diseases such as Duchenne muscular dystrophy, cerebral palsy, and hereditary spastic paraplegia were suspected until the older sibling reached the age of seven. However, the presence of the condition was confirmed through genetic screening.

The rehabilitation program was developed to address the main issues of the cases. Both participants were attending school and aimed for academic success. Moreover, the families’ expectation was for their children to lead as independent a life as possible. Particularly in terms of joining friends at school, problems arose due to balance and coordination difficulties. In developing the rehabilitation program, we took into account other pediatric neurological studies and the typical symptoms experienced by our patients [16-18].

The significance of maintaining trunk control for balance is crucial for both adults and children [19]. Trunk control is an important milestone that supports all rehabilitation processes, particularly in children affected by neuromuscular disorders such as cerebral palsy [20]. Postural control in children with cerebral palsy worsens in comparison to their typically developing peers, potentially due to delayed and impaired development of neural motor control mechanisms and common secondary musculoskeletal abnormalities. After 18 weeks, we have made progress in parameters relating to balance and independence, a result of implementing a program focused on balance and coordination development. Although not evaluated in the study, the family reported a decrease in falls and improved running speed for both siblings following the treatment. We believe that this is closely linked to the use of balance exercises that involve trunk control.

The study had several limitations. The rehabilitation facility was used by patients and physiotherapists concurrently. This made it challenging to hold the attention of patients during specific exercises. Additionally, varying motivations among the patients represented a significant limitation. For instance, the older brother was resistant to implementing the rehabilitation program, despite having more severe issues. Taking breaks during exercise is vital for this reason. This study is a case report, and further testing of the methods is necessary in evidence-based, randomized studies involving multiple participants.

## Conclusions

The purpose of the investigation was to evaluate the impact of an 18-week rehabilitation program, comprising balance and coordination exercises, on two siblings diagnosed with neurodegeneration caused by iron accumulation in the brain. Comprehensive understanding of these conditions is imperative to determine the most appropriate therapy. Hence, balance and coordination-based exercise regimes have become a vital element of clinical intervention for patients affected by MPAN syndrome. It is crucial that rehabilitation programs are tailored according to the fundamental requirements of the patients or their families. The 18-week rehabilitation program for MPAN syndrome in this study has beneficial effects on muscle strength, balance parameters, and independence.
